# Benchmark for Peak Detection Algorithms in Fiber Bragg Grating Interrogation and a New Neural Network for its Performance Improvement

**DOI:** 10.3390/s110403466

**Published:** 2011-03-24

**Authors:** Lucas Negri, Ademir Nied, Hypolito Kalinowski, Aleksander Paterno

**Affiliations:** 1 Department of Electrical Engineering, Santa Catarina State University (UDESC), Joinville 89223-100, Brazil; E-Mails: lucashnegri@gmail.com (L.N.); dee2an@joinville.udesc.br (A.N.); 2 Graduate School of Electrical Engineering and Computer Science, Federal University of Technology-Paraná (UTFPR), Curitiba 80230-901, Brazil; E-Mail: hjkalin@cpgei.ct.utfpr.edu.br

**Keywords:** fiber Bragg grating, optical sensing, peak detection, fitting, optimization

## Abstract

This paper presents a benchmark for peak detection algorithms employed in fiber Bragg grating spectrometric interrogation systems. The accuracy, precision, and computational performance of currently used algorithms and those of a new proposed artificial neural network algorithm are compared. Centroid and gaussian fitting algorithms are shown to have the highest precision but produce systematic errors that depend on the FBG refractive index modulation profile. The proposed neural network displays relatively good precision with reduced systematic errors and improved computational performance when compared to other networks. Additionally, suitable algorithms may be chosen with the general guidelines presented.

## 1. Introduction

Fiber Bragg grating (FBG) interrogation techniques now form a mature field of research where computational techniques must be used to improve the process of monitoring FBG sensors. When used as sensors, FBG are usually subject to uniform fields of certain types of perturbation such as temperature or strain. In this case, the spectrum of the light reflected by the sensor has its peak monitored, indicating the magnitude of the perturbation. Many techniques which are also part of commercial systems use a periodically tunable laser source to illuminate the FBG and produce the signal corresponding to the spectrum of the light that interacts with the device [1], characterizing the spectrometric technique. These wavelength sweeping techniques may have high accuracy and precision, requiring an additional computational processing of the acquired signals, and a wavelength signal reference which is systematically used during the sensor interrogation process [2]. Computational intelligence algorithms have been used to improve the peak detection accuracy in signals with distorted spectrum in FBG strain sensors. This process provided an improvement in peak detection accuracy in a signal with noise and distortion caused by a non-uniform disturbance in the sensor. One drawback of any neural network use is its time execution performance which might be long to the implementation in embedded hardware. Other neural networks were also proposed that could provide equivalent performance in terms of accuracy and precision. In such cases, the approximation occurs during the training phase of the neural network and is applied whenever the peak identification is necessary [3,4]. Other simpler techniques demonstrate similar performances in terms of uncertainty and precision, but are not capable of dealing with the same type of distortion solved by the neural network approach. More typical techniques are based on least-squares (LS) fitting algorithms of an FBG spectrum, which result in acceptable performance in interrogation systems as reported in the literature [5]. The use of a simple algorithm to find the centroid of a spectrum profile would also provide useful results [6].

A benchmark evaluation of algorithms used in peak detection must be provided to produce guidelines to evaluate the performance of the algorithms, including the least-squares fitting, computational intelligence and simpler techniques. The benchmark for peak detection algorithms in the interrogation of FBG sensors and the adapted algorithms are therefore made publicly available for general applications [7]. In addition to the addressed problems, some aspects of the computational complexity of neural networks algorithms for application in FBG interrogation are also evaluated by proposing the use of a new computationally efficient training algorithm for neural networks. This may establish the limit in the time execution performance of a neural network used in FBG interrogation with the additional benefit of keeping the neural network performance of detecting the peak under different conditions of distortion and noise in the spectrum signal.

## 2. Methodology

For comparison purposes, a brief description of the most frequently used algorithms to detect peak position in the FBG spectrum will be provided in the following subsections. For testing purposes the spectrum signal is simulated with different levels of additive white Gaussian noise (AWGN). The algorithms were also applied in experimental data obtained with a tunable laser illuminating FBG sensors. The proposal of the neural network algorithm for the approximation of the FBG spectrum is described and the determination of its peak position statistics is then calculated, as equivalently implemented for the other algorithms.

The described algorithms use input spectra with normalized amplitudes from 0 to 1 (0% and 100% reflectivity, respectively). The transfer matrix method [8] is used to generate the test spectra for the FBG with uniform and gaussian index modulation [2] profiles with a resolution of 0.1 pm. The mean refractive index change used was 10*^−^*^4^ for the FBG with a length of 5 mm in a standard single-mode fiber. The simulated spectrum amplitude was normalized. The wavelength peak position of each spectrum is determined by the maximum amplitude value position, and its resolution is then reduced to 1 pm (wavelength distance between two consecutive points) and white-Gaussian noise is added to the simulated signal with different signal-to-noise ratios (SNR). The signals were generated for SNR values of 16 dB up to 60 dB in steps of 2 dB. Sets of 300 input data for each SNR, using the same spectrum as a signal, were generated to determine the statistics of the peak detection error.

The peak detection algorithms listed below were tested and their characteristics were evaluated with the simulated spectra, verifying the accuracy (the mean of the difference between the peak obtained in a noisy spectrum and the noiseless peak in a high resolution spectrum) and the precision (*σ*, sample standard deviation of the error) of each algorithm for each spectra set related to an SNR level.

The computational performance was verified for a sample spectrum, measuring the time needed for the computation of each algorithm implementation using an input signal with an SNR of 60 dB. The implementations were compiled by *gcc*, version 4.5.1 and tested with an *Intel^®^ Core^™^2 Duo T7300* processor.

To measure its performance, the proposed neural network training algorithm was also compared to other currently used algorithms in neural networks having the same topology and configuration as the proposed algorithm,, namely the back-propagation [9] and iRprop [10] algorithms. For the evaluation of time execution performance, the algorithms were executed until the network reached a mean square error (MSE) of 0.0015 or smaller, or performed 10^6^ iterations (epochs), whichever came first. The algorithms were tested using a spectrum from an FBG with gaussian apodization and an SNR of 60 dB.

### 2.1. Test with Experimental Data

**Figure 1.**
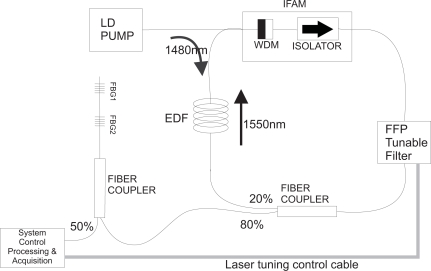
System schematic diagram for the interrogation of FBG sensors, FBG1 and FBG2, with an Erbium-doped fiber laser (EDFL), a tunable Fabry-Perot filter, EDF in a ring cavity and electronic tuning control.

The tunable laser for the interrogation system is an Erbium-doped fiber (EDF) ring laser with an intra-cavity Fabry-Perot filter (FPF) whose schematic design is depicted in Figure 1. The signal produced by the laser illuminates fiber Bragg grating sensors through a fiber coupler. The reflected signal is acquired with a photo-detector circuit and sent to a processing unit, which also controls the laser tuning. The EDF has a length of 17 m with Er concentration of 280 ppm. The pump power of approximately 70 mW is introduced inside the EDF through a WDM coupler, which is part of an IFAM (Integrated Fiber Amplifier Module). The optical isolator, also integrated with the WDM coupler, prevents the light to propagate bi-directionally in the cavity. Through the output coupler, approximately 80% of the laser light is emitted and the other 20% are used for optical feedback. The tuning range of the FPF (TB2500, JDS FITEL) can be set by means of a voltage applied to its terminals which are connected to the PZT that moves the mirrors of the filter. By correctly setting the voltage range to the FFP, the laser can be tuned from 1,525 to 1,565 nm at the room temperature of 24 °C during the experiment. Between these wavelengths the intensity of the laser output is approximately constant and higher than −20 dBm and its line-width is also constant with an estimated value of 67 pm measured with an all-fiber interferometer.

**Figure 2.**
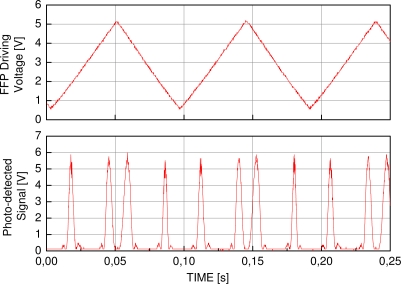
Representation of the tuning waveform and the photo-detected signal corresponding to the two FBG sensors. The signal is time-based and must be converted to wavelength based on the FBG sensors Bragg wavelength.

The experimental data were acquired by a photo-detecting circuit collecting the light reflected by the sensors, which were illuminated by the EDFL sensor interrogation system. Two sensors were periodically monitored at a sweeping frequency of 10 Hz along a wavelength interval of 10 nm. A triangular waveform with a 50% duty-cycle was used as the tuning signal in the laser, while the processing of the acquired signal could be applied during the fall time of the triangular tuning waveform. The data is composed of 20 measurements, where the signal is acquired by a photo-detector circuit during the rise time of the triangular waveform that sweeps the laser wavelength. The acquired data comprise a signal containing the information from the two FBGs, the sensor and the reference FBG, with Bragg wavelength at 1,542.9 nm and 1,547 nm. One of the FBG sensors is connected to a mechanical apparatus able to stretch it in a controlled manner and its length is measured by a micrometer. The other FBG is kept unperturbed and close to the perturbed sensor to compensate temperature variations during the characterization. An example of the tuning signal and the photo-detected signal from the sensors is depicted in Figure 2. In this test, the time difference between the peak positions corresponding to the sensor and the reference are computed for each strain level. Since the signal is time-based, the time axis must be converted to wavelength using the reference peak.

Another test verified the usage of the algorithms for peak detection of experimental data. The experimental data were acquired with an FBG sensor interrogation system based on a tunable Erbium-doped fiber laser. Two sensors were periodically monitored at a sweeping frequency of 10 Hz along a wavelength interval of 10 nm. A triangular waveform with a 50% duty-cycle was used as the tuning signal in the laser, while the processing of the acquired signal could be applied during the fall time of the triangular tuning waveform. The data is composed of 20 measurements, where the signal is acquired by a photo-detector circuit during the rise time of the triangular waveform that sweeps the laser wavelength. The acquired data comprise a signal containing the information from the two FBGs, the sensor and the reference FBG, with Bragg wavelength at 1,542.9 nm and 1,547 nm. One example of the tuning signal and the photo-detected signal from the sensors is depicted in Figure 2.

Similar to the simulated data, the algorithms were evaluated with respect to their accuracy and precision. Seeing that the data is experimental there is no expected peak to calculate the accuracy statistics. Since the relation between strain and peak shift is expected to be linear, the accuracy of the algorithms is defined here as the mean square error between the obtained points in the experimental setup and the linear fitted line.

Simulated AWGN were added to a base data set that already incorporates noise, generating spectra with additional SNR values of 16 dB up to 60 dB in steps of 2 dB. Each SNR group is composed by 100 data sets, each formed by 20 spectra, one spectra per strain. The accuracy computed by the previously exposed method is presented for each SNR along with the precision (sample standard deviation instead of the mean value).

## 3. Peak Detection Algorithms

**Figure 3.**
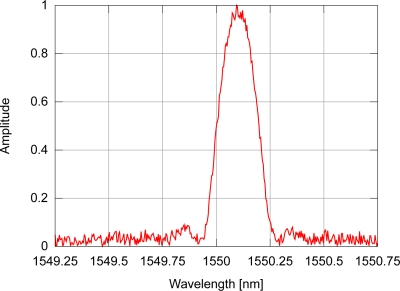
Example of optical spectrum for an FBG with uniform modulation index profile and AWGN.

**Figure 4.**
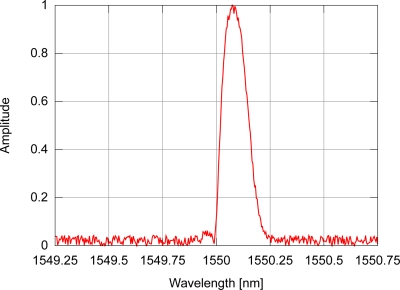
Example of optical spectrum for an FBG with gaussian modulation index profile and AWGN.

If the FBG sensor is subjected to uniform disturbances along its length, the sensor’s modulation index profile will be uniformly altered; this causes the FBG reflectivity spectrum to be uniformly shifted towards lower or higher wavelengths, meaning that any point close to the spectrum peak will determine how the sensor behaves. Due to intrinsic characteristics of the hardware in interrogation systems, the spectrum can incorporate noise and the actual peak wavelength may differ from the peak found in a simple search for the highest value in the photo-detected signal, making it necessary to have suitable peak detection algorithms. In a fiber Bragg grating sensor, the spectrum profile of the light reflected by the sensor also depends on the refractive index modulation profile in the fiber optic core. Due to specific fabrication process characteristics, the modulation index envelope may not be uniform. To explore the behavior of the algorithms, two different spectra will be used: one with symmetrical side-lobes around the spectrum peak (uniform index modulation); and the other with non-symmetrical side-lobes produced by an FBG with a gaussian modulation index profile. An example of a simulated FBG spectrum resulting from the light reflected by a sensor with uniform modulation index profile and signal-to-noise ratio of 30 dB (SNR) is depicted in Figure 3, while Figure 4 shows the spectrum of the light reflected by a sensor with gaussian modulation index profile. Both signals include AWGN.

### 3.1. Maximum

The maximum algorithm is based on the search for the wavelength with the highest amplitude in the input data. This method is used as a reference of time execution performance but not of accuracy and precision, due to the naturally high inherent noise sensitivity.

### 3.2. Discrete-Time Filter

This algorithm uses a linear phase finite impulse response (FIR) low-pass filter to attenuate the high frequency noise and then uses the maximum algorithm to find the peak. This algorithm is designed to have a relatively low order and complexity. The low-pass filter was designed based on a Fourier analysis of the noiseless spectrum signal from uniform and gaussian modulation profiles of FBG sensors. It is an equiripple FIR filter with a normalized passband cutoff frequency at the first zero crossing point of the sensor signal Fourier transform. The FIR filter has an attenuation stopband of 80 dB. This resulted in a filter with order *M* = 36. Time domain convolution was used to filter the sensor signal since *M* was sufficiently small to justify its use instead of a frequency domain filtering technique. With respect to the used input data, to enhance the computational performance those points whose amplitude is lower than 0.4 are discarded.

### 3.3. Centroid

The centroid algorithm produces a point corresponding to the geometric centroid of a spectrum, calculated by Equation 1, where *N* is the size of the spectrum points vector, *λ_i_* is the *i*-th point wavelength, and *A_i_* is the *i*-th point amplitude. This method has already been used in other works [6,11].

(1) $$\lambda_{b}=\frac{\sum_{i=1}^{N}\lambda_{i}A_{i}}{\sum_{i=1}^{N}A_{i}}$$

In this algorithm, the spectrum centroid determines how the spectrum is being shifted. Before being fed to the centroid, the input spectrum is centered by removing those points with amplitude lower than 0.4.

### 3.4. Least Squares Fitting

Another currently used peak detection algorithm consists of adjusting models to fit the spectrum. With fiber Bragg grating sensors it is natural to use a gaussian or a polynomial function as a model [6], since such functions may well approximate at least the peak region of the spectrum of the light reflected by an FBG.

In this work, the gaussian fitting is implemented by minimizing the squared errors using the Gauss-Newton algorithm. The adjusted gaussian function is shown in Equation 2, where *A*, *C*, and *V* are the adjusted parameters (amplitude, center, and deviation) and *y_i_* is the calculated amplitude for the *λ_i_* wavelength.

(2) $$y_{i}=A\;\text{exp}\left(-\frac{{\left(C-\lambda_{i}\right)}^{2}}{{2V}^{2}}\right)$$

**Figure 5.**
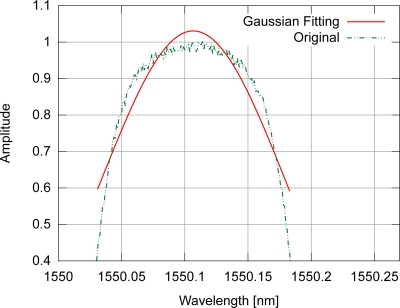
Example of gaussian fitting for a spectrum from an FBG with uniform modulation index profile.

As shown in Figure 5, the gaussian fitting of an optical spectrum from an FBG with uniform modulation does not result in a signal with the same shape, but there is a correlation between their peaks, which makes this procedure interesting for peak detection in FBG sensors. For the gaussian fitting, only those points with amplitude equal to or greater than 0.4 were used.

The polynomial fitting was also implemented using the Gauss-Newton algorithm. A third order polynomial was used (Equation 3 with *n* equal to 3), where *n* is the order, *y_i_* is the calculated amplitude for point *i*, *λ_i_* is the wavelength of point *i*, and *c_j_* are the polynomial coefficients. Since the derivative of Equation 3 with respect to a specific coefficient *c_j_* is a constant, the system is said to be linear and only one iteration of the Gauss-Newton algorithm is needed to optimize the coefficients [12].

(3) $$y_{i}=\sum_{j=0}^{n}c_{j}\lambda_{i}^{j}$$

**Figure 6.**
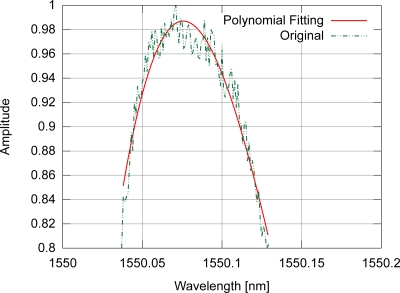
Example of polynomial fitting for a spectrum from a gaussian apodized FBG.

In the polynomial fitting, the amplitudes of the input spectrum are also normalized between 0 and 1, discarding the points with amplitude lower than 0.8. An example of polynomial fitting for a spectrum with gaussian modulation is shown in Figure 6.

### 3.5. Neural Network

**Figure 7.**
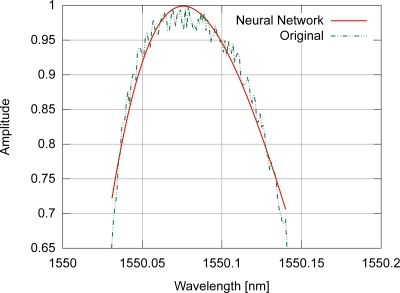
Example of neural network fitting for a spectrum from a gaussian modulated FBG.

A properly constructed artificial neural network is a universal function approximator, as seen in the literature [13–15]; in a previous work [4] an ADALINE neural network was employed for peak detection, removing unwanted interferometric signals. These facts contributed to the proposal of this new peak detection algorithm, using a fully connected cascade (FCC) artificial neural network [16], but now the computational performance may be improved, with the additional advantage of maintaining the capacity of the algorithm to correct some types of distortions in the approximated spectrum. Similar to the gaussian and polynomial fittings, the neural network tries to approximate the general shape of the target function, ignoring the noise. In addition the algorithm allows the use of symmetrical and non-symmetrical profiles without causing more pronounced systematic errors in the peak determination process. The unwanted noise approximation is handled by using a reduced number of neurons and synaptic connections, preventing such over-fitting to occur. As an example, Figure 7 depicts the fitting of a noisy spectrum with the proposed algorithm. Depending on the training method of the neural network, different techniques may be used to relax the training phase, avoiding noise fitting [3].

**Figure 8.**
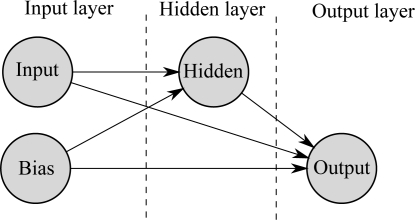
Topology of the proposed FCC neural network.

The proposed neural network is composed of four neurons, disposed in three layers, as shown in Figure 8. The first layer comprises two neurons, one being the input neuron and the other a bias neuron, with the second and third layers having one neuron each. All neurons have forward connections with every neuron in the next layers, resulting in a total of 5 synaptic connections. The connection between the neuron in the second layer and the output neuron uses a sigmoid activation function, while the output neuron uses a linear activation function.

The network was trained by the *Neuron by Neuron* (NBN) algorithm [16], using a previously implemented and publicly available library [7]. The NBN algorithm consists of a performance optimization of the Levenberg-Marquardt algorithm [17]. When applied in neural network training, the LM algorithm adjusts the synaptic weights of the trained network using Equation 4, where *w* is the weight matrix, *H* is the Quasi-Hessian matrix, *G* is the error gradient and *μ* is a normalization factor.

(4) $$\Delta w={\left(H+\mu I\right)}^{-1}G$$

For the proposed neural network, the Quasi-Hessian matrix is calculated by Equation 5, where *J* is a *m* × *n* Jacobian matrix and *J^T^* is its transpose, with *m* equal to the number of data points (wavelength-amplitude tuple) and *n* equal to the number of synaptic weights. Each data point results in one row of the Jacobian matrix, calculated by back-propagation.

(5) $$H=J^{T}J$$

The error gradient is calculated by Equation 6, with the error matrix *E* calculated by forward propagation.

(6) $$G=J^{T}E$$

Due to its size, which is a function of the number of data points, the full Jacobian matrix needs more memory to be stored than the *H* and *G* matrices. The optimization resulting from the NBN algorithm consists of building *H* and *G* directly, without storing the full Jacobian matrix [16]. This significantly reduces the memory needed by the training, since both the Quasi-Hessian and gradient matrices sizes depend only on the number of synaptic weights, which is usually more than one order of magnitude smaller than the number of data points. Although this optimization does not reduce the computational complexity of the algorithm, it results in performance gains due to reduced memory allocations.

The optimization introduced by NBN is based on the fact that matrix multiplication can be implemented by multiplying the columns of the left operand with the rows of the right operand, resulting in a summation of partial matrices, that can be added to a result matrix as soon as they are calculated. As both the Quasi-Hessian and gradient matrices equations involve the transposed Jacobian matrix as left operand, one can calculate them iteratively, after the calculation of each row of the Jacobian matrix.

Only those data points with amplitude equal to or greater than 0.65 were used, with the wavelengths scaled between 0 and 1 to match the activation function operation range. Additionally, the method converged faster when initializing all the weights with random positive values.

### 3.6. Results

**Figure 9.**
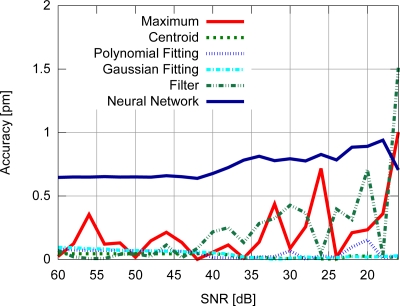
Accuracy for the uniform modulation profile.

**Figure 10.**
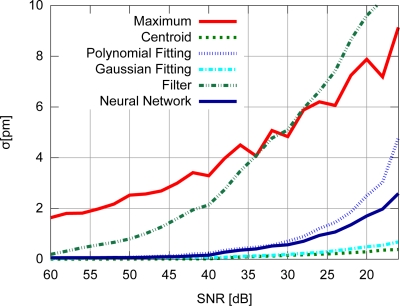
Precision for the uniform modulation profile.

**Figure 11.**
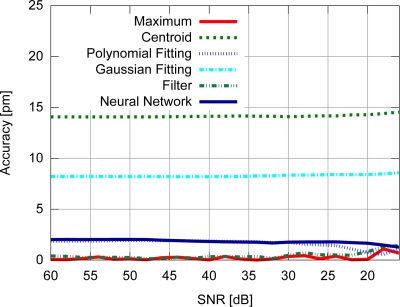
Accuracy for the gaussian modulation profile.

**Figure 12.**
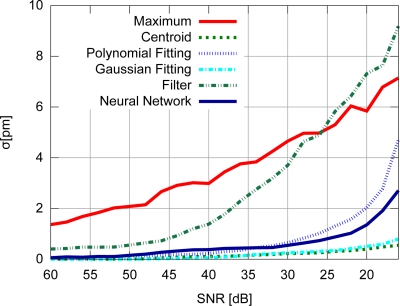
Precision for the gaussian modulation profile.

Accuracy results for the FBG with uniform modulation profile are shown in Figure 9, while the corresponding precision values are shown in Figure 10. For the gaussian modulation profile, the accuracy results appear in Figure 11, with precision in Figure 12.

The computational performance of the algorithms is also a critical factor to provide a guideline for the implementation of the algorithm in a real-time system or in embedded hardware. Table 1 shows the algorithms execution performance, using a normalized factor, *i.e.*, all values are normalized using the execution time of the maximum algorithm as a reference. For reference purposes, the maximum algorithm runs in approximately 0.5 *μ*s on the test hardware.

### 3.7. Experimental Results

**Figure 13.**
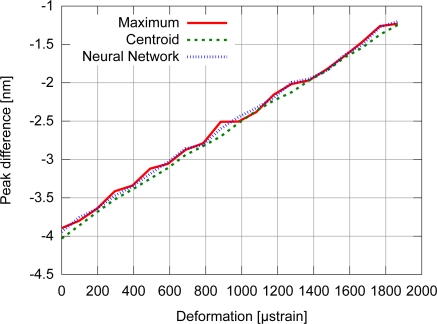
Relation between strain and spectrum position by different peak detection algorithms.

The FBG strain sensor shows a linear response, as the perturbation was within the physical strain limit of the sensor. Examples of experimental calculated points corresponding to the wavelength difference between the reference FBG peak and the strain sensor peak, as a function of the strain level, are depicted in Figure 13. Three of the algorithms were chosen based on their characteristics of execution time, uncertainty and precision, for which the fluctuation of the points would show the behavior of the algorithm under practical conditions. The chosen algorithm could be used if its execution time was shorter than the fall time of the tuning triangular waveform in the tunable laser.

The values for the different neural network training algorithms are shown in Table 2, where the resulting mean MSE and mean epochs needed to reach any of the stopping conditions are presented. As an example, both the iRprop and Incremental algorithms failed to reach the target MSE, reaching the epoch limit.

## 4. Discussion

With respect to the FBG signal corresponding to the sensor with uniform modulation profile, the results presented in Figures 9 and 10 support the conclusion that the gaussian fitting and the centroid are more accurate and precise than the other algorithms, and have a relatively good noise tolerance. As expected, the naïve maximum algorithm shows a higher standard deviation (lower precision). The polynomial fitting and neural network algorithms demonstrated similar precision, although the neural network has shown a higher peak difference for different levels of SNR. The filter has shown better results than the maximum algorithm until 30 dB, with worse results for lower SNR levels. This indicates that the deteriorated precision level while using the filter to improve peak detection is caused by low frequency noise which could not be suppressed by the filtering process. However for practical SNR levels above 30 dB, the filtering may be used.

The results presented in Figures 9 and 10 also support the conclusion that the centroid and gaussian fittings have the highest precision (lowest deviation) between the algorithms, although they also produce systematic errors. In fact, the precision of the algorithms for the gaussian profile are similar to the uniform profile. For the FBG sensor with a gaussian index modulation profile, the proposed neural network algorithm has also shown a systematic deviation in the accuracy, but less than what was observed in the centroid and gaussian fitting errors. Even when considering the lack of symmetry in the signal obtained from a Gaussian-apodized FBG, the precision and accuracy did not show much difference, and could be considered similar for different spectrum profiles. This is only evidenced when calculating the statistics with more than 100 input data spectra for each *SNR*. Considering the gaussian-apodized FBG, the systematic error produced by the centroid algorithm is related to the spectrum asymmetry, while the error produced by the gaussian fitting is related to the mismatch between the model being fitted and the spectrum shape.

**Figure 14.**
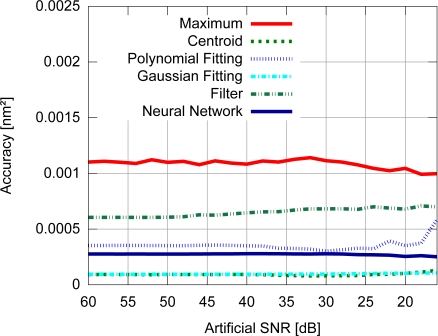
Accuracy for the experimental data test calculated using the mean square error between measured points and the fitted straight line.

**Figure 15.**
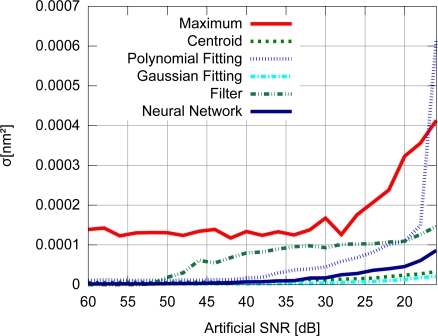
Precision for the experimental data test based on the square error standard deviation based on the measured points and the fitted straight line.

The results of the experimental data test presented in Figures 14 and 15 showed a similar behavior for both simulated and experimental data, in spite of the test methodology and unit differences. Due to the usage of a reference sensor, the systematic errors shown by the centroid and gaussian algorithms in the simulations could be canceled, causing these two algorithms to have the best accuracy among the evaluated algorithms.

The computational performance of the algorithms differs by orders of magnitude, preventing the usage of some algorithms in applications that require superior performance. As seen in Table 1, the maximum and centroid algorithms have the best performance. Even though they have the same computational complexity when considering the vector input size, the centroid performs better due to implementation details. The filter method is approximately one order of magnitude slower than the maximum algorithm, followed by the polynomial and gaussian fittings. The neural network algorithm had the worst performance: approximately four orders of magnitude slower than the maximum algorithm using the highly optimized Neuron-by-Neuron algorithm. However, as shown in the literature, such neural network algorithms may solve spectrum distortion problems caused by noise and non-uniform disturbance in the sensors [3]. Additionally, the training algorithm used by the proposed neural network has demonstrated significant enhancements over currently used algorithms, as shown in Table 2 and established a lower limit in time performance for use of the neural network in this type of FBG interrogation.

When considering the practical implementation of this training algorithm or the trained neural network, a specific processor would be required for the implementation of the previously mentioned matrix calculation algorithms, otherwise the algorithm will not be capable of operating in a real-time manner. Hardware implementation of neural network algorithms or its components may be a direction to the practical implementation of the computationally intelligent signal processing in the interrogation, since there is a current trend to implement neural algorithms in dedicated hardware, for example, using modular neural networks (MNN) [18]. The use of digital signal processors with the neuron-by-neuron algorithm and optimized libraries for the matrix calculations would also be a straightforward suggestion. The criteria to select the neural network instead of the centroid or the gaussian fitting would be based on the application and how much information the user would like to extract from the processed data.

One strategy to enhance the performance of the neural network algorithm for specific cases is to initialize the weights with previously calculated values, where the network training will be responsible only for the fine-tuning of the weights, performed with a smaller number of training iterations (epochs).

For the FBG sensor with a uniform index modulation profile and the FBGs employed in the experimental data test, it is clear that the centroid and gaussian fitting algorithms have advantages over the other algorithms, due to their higher accuracy, precision and computational performance. However, it is not possible to conclude the same for the gaussian profile. Regarding the FBG with a gaussian index modulation profile, the optimum algorithm choice depends on application circumstances such as the expected SNR in the optoelectronic system, and the performance requirements. However, the centroid and parametric fitting methods can still give good results with applications where the systematic error can be corrected or does not matter.

Due to the longer training phase time to apply the algorithms in experimental data, ordinary neural network training algorithms could be used only in off-line processing of the signal, and not at such tuning frequencies in the laser with this interrogation technique. The largest differences between the fitted line and the measured processed experimental points were obtained for the maximum algorithm. The neural network algorithm shows a smaller amplitude fluctuation than the maximum algorithm, and as previously shown the centroid demonstrates the smallest fluctuations.

## 5. Conclusions

In this work a benchmark for algorithms used in FBG interrogation systems was implemented. A new algorithm for training a neural network used as a universal approximator of the FBG reflective spectrum was proposed, and its performance was compared with other algorithms used for the same purpose. The Neuron-by-Neuron training algorithm improves the time performance of the neural networks used to approximate the FBG spectrum. Due to the capacity of neural networks to correct some distortions in the spectrum, they could be used as an alternative to more elementary algorithms. In addition, this training algorithm may establish a lower limit in the time performance enhancement when using neural networks to approximate FBG spectrum, allowing them to be used in real-time processing and embedded systems. As a general rule, the centroid may be considered the fastest and most precise algorithm, even when producing a larger systematic error due to the eventual occurrence of non-symmetrical spectrum signals. This benchmark also provides some guidelines for researchers to choose the proper algorithm for their own application.

## Figures and Tables

**Table 1.** Computational performance of the evaluated implementations.

Algorithm	Normalized time
Maximum	1
Centroid	0.67
Filter	27
Polynomial Fitting	340
Gaussian Fitting	943
Neural Network	25,000

**Table 2.** Performance of different neural network training algorithms.

Training Algorithm	Mean MSE	Mean Epochs
NBN	1.45	11
iRprop	1.6	10^6^
Incremental	3.5	10^6^
